# Supplementary material for: Alterations in the Kynurenine–Tryptophan Pathway and Lipid Dysregulation Are Preserved Features of COVID-19 in Hemodialysis
Source: Int J Mol Sci. 2022 Nov 15;23(22):14089. doi: 10.3390/ijms232214089 (PMC9698708; doi:10.3390/ijms232214089)
Supplement: Supplementary file 1 [file ijms-23-14089-s001.zip › ijms-2002086-supplementary.pdf]

SUPPLEMENTARY MATERIALS AND METHODS – TABLE OF CONTENT

		Page number
**Figure S1**	Disturbances of the arginine and proline pathway and purine metabolism in COVID-19	2
**Figure S2**	Distinctively deregulated metabolites in moderate COVID-19 between HD and non-HD	3
**Figure S3**	Creatinine levels and association of creatinine with markers of moderate COVID-19 in non-HD	4

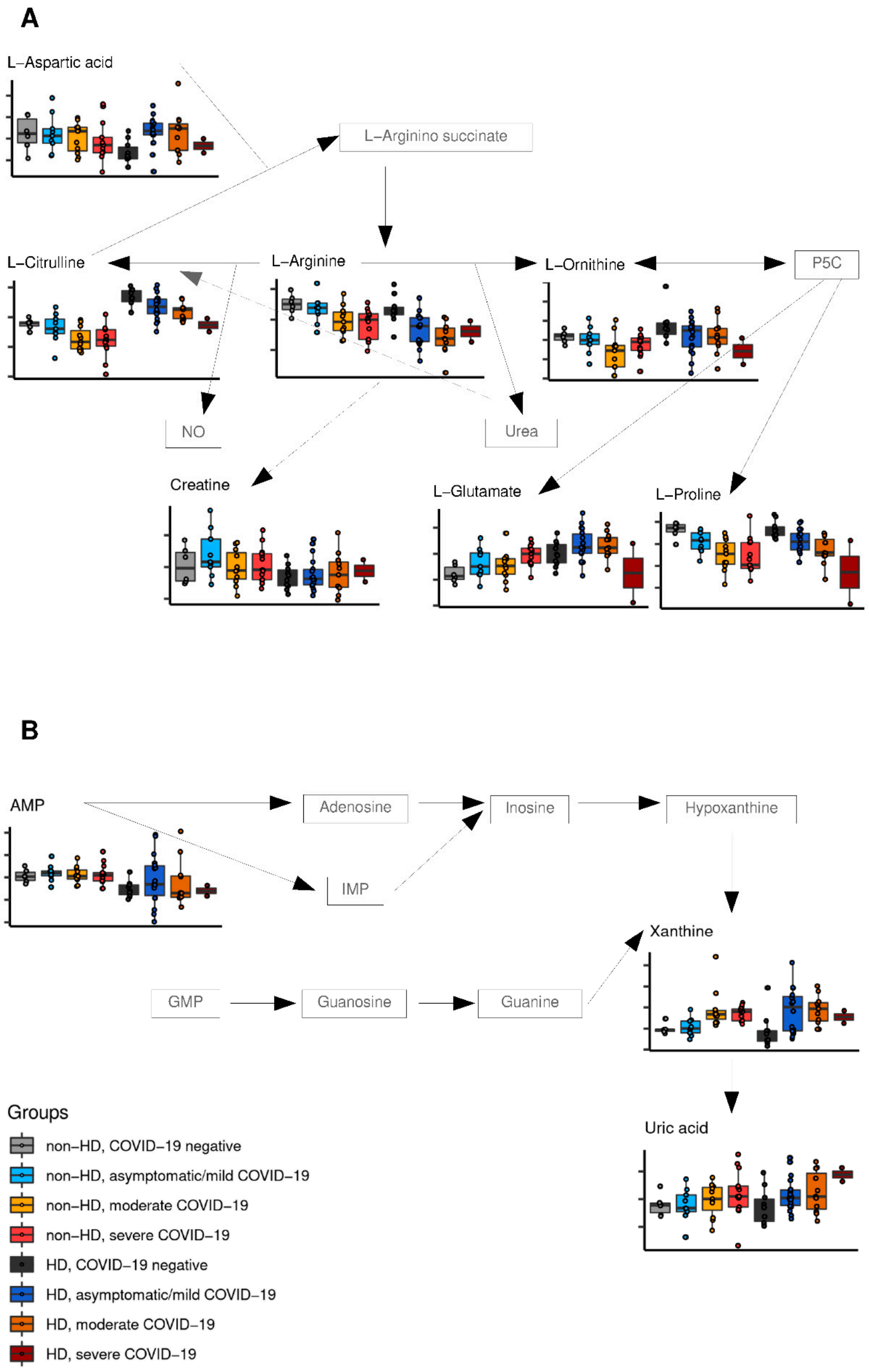
A
L–Aspartic acid
L–Arginino succinate
L–Citrulline
L–Arginine
L–Ornithine
P5C
NO
Urea
Creatine
L–Glutamate
L–Proline
B
AMP
Adenosine
Inosine
Hypoxanthine
IMP
Xanthine
GMP
Guanosine
Guanine
Uric acid
Groups
non–HD, COVID–19 negative
non–HD, asymptomatic/mild COVID–19
non–HD, moderate COVID–19
non–HD, severe COVID–19
HD, COVID–19 negative
HD, asymptomatic/mild COVID–19
HD, moderate COVID–19
HD, severe COVID–19

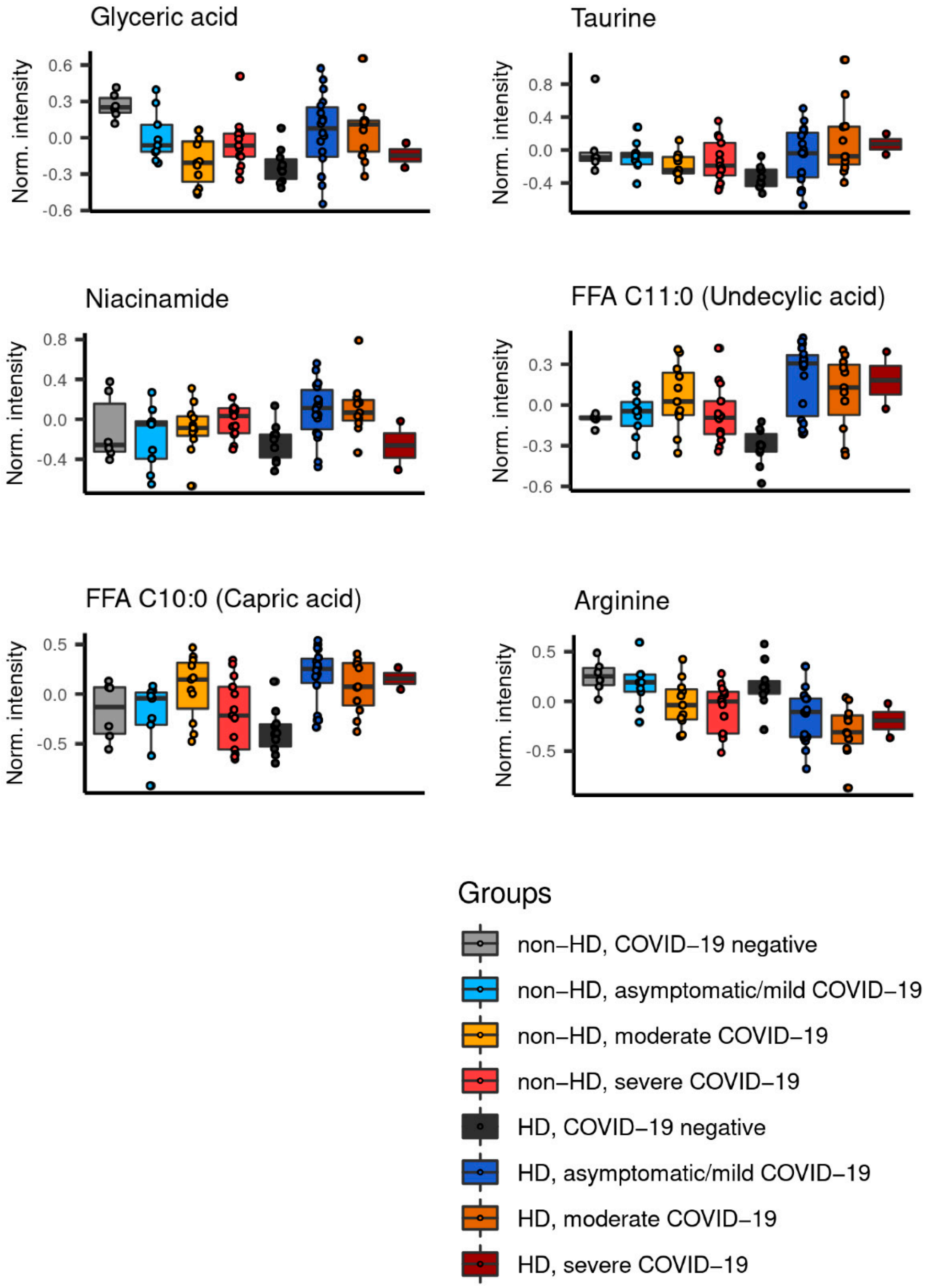
Glyceric acid
Taurine
Niacinamide
FFA C11:0 (Undecylic acid)
FFA C10:0 (Capric acid)
Arginine
Norm. intensity
Groups
non–HD, COVID–19 negative
non–HD, asymptomatic/mild COVID–19
non–HD, moderate COVID–19
non–HD, severe COVID–19
HD, COVID–19 negative
HD, asymptomatic/mild COVID–19
HD, moderate COVID–19
HD, severe COVID–19

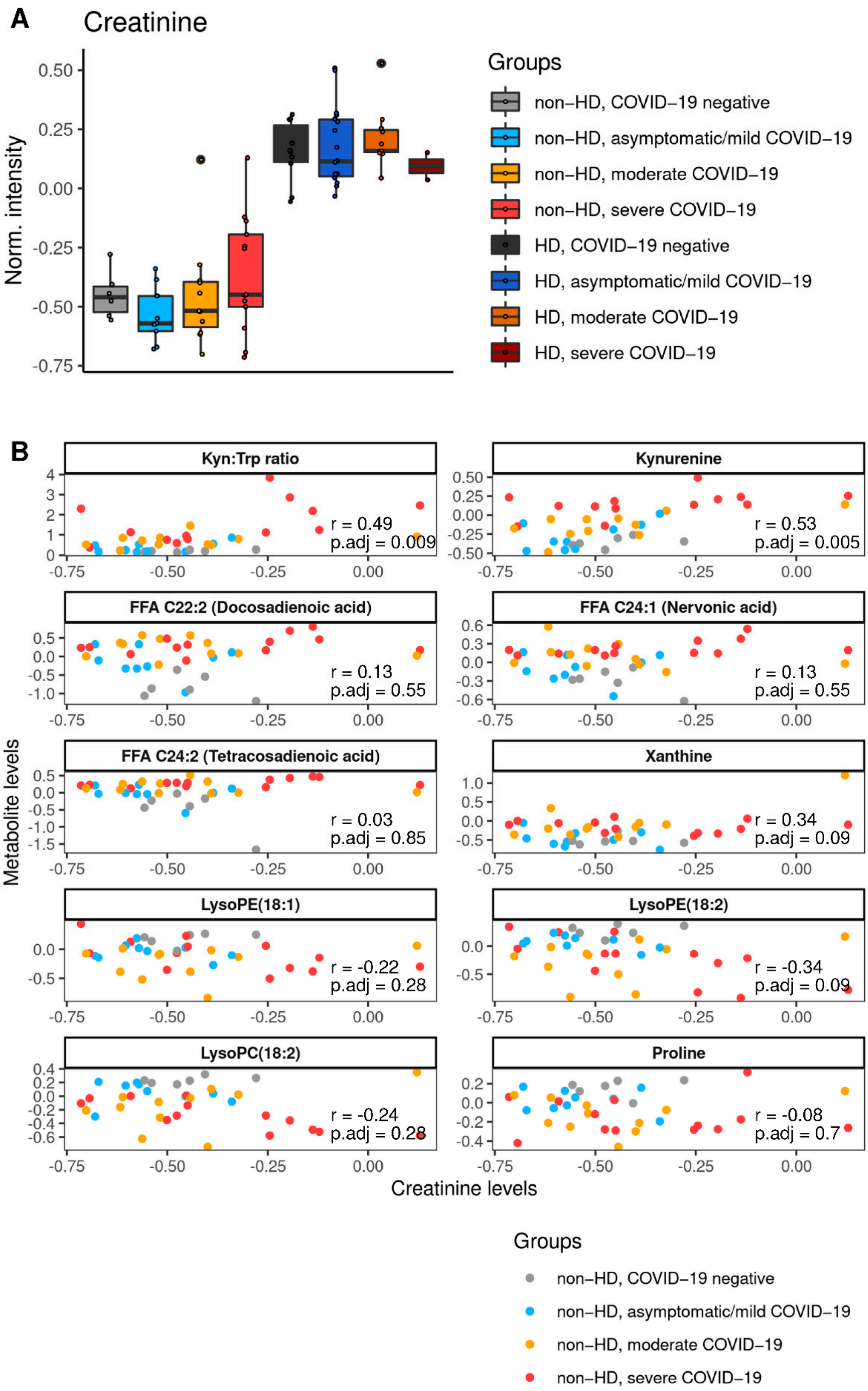
A
Creatinine
Norm. intensity
Groups
non–HD, COVID–19 negative
non–HD, asymptomatic/mild COVID–19
non–HD, moderate COVID–19
non–HD, severe COVID–19
HD, COVID–19 negative
HD, asymptomatic/mild COVID–19
HD, moderate COVID–19
HD, severe COVID–19
B
Kyn:Trp ratio
r = 0.49
p.adj = 0.009
Kynurenine
r = 0.53
p.adj = 0.005
FFA C22:2 (Docosadienoic acid)
r = 0.13
p.adj = 0.55
FFA C24:1 (Nervonic acid)
r = 0.13
p.adj = 0.55
FFA C24:2 (Tetracosadienoic acid)
r = 0.03
p.adj = 0.85
Xanthine
r = 0.34
p.adj = 0.09
LysoPE(18:1)
r = -0.22
p.adj = 0.28
LysoPE(18:2)
r = -0.34
p.adj = 0.09
LysoPC(18:2)
r = -0.24
p.adj = 0.28
Proline
r = -0.08
p.adj = 0.7
Metabolite levels
Creatinine levels
Groups
non–HD, COVID–19 negative
non–HD, asymptomatic/mild COVID–19
non–HD, moderate COVID–19
non–HD, severe COVID–19
